# *BSG* and *MCT1* Genetic Variants Influence Survival in Multiple Myeloma Patients

**DOI:** 10.3390/genes9050226

**Published:** 2018-04-24

**Authors:** Piotr Łacina, Aleksandra Butrym, Grzegorz Mazur, Katarzyna Bogunia-Kubik

**Affiliations:** 1 Laboratory of Clinical Immunogenetics and Pharmacogenetics, Hirszfeld Institute of Immunology and Experimental Therapy, Polish Academy of Sciences, 53-114 Wrocław, Poland; bogunia@iitd.pan.wroc.pl; 2 Department of Internal, Occupational Diseases, Hypertension and Clinical Oncology, Wroclaw Medical University, 50-556 Wrocław, Poland; aleksandra.butrym@gmail.com (A.B.); grzegorz.mazur@umed.wroc.pl (G.M.)

**Keywords:** basigin, monocarboxylic acid transporter 1, single nucleotide polymorphisms, multiple myeloma, survival

## Abstract

Multiple myeloma (MM) is a haematologic malignancy characterized by the presence of atypical plasma cells. Basigin (BSG, CD147) controls lactate export through the monocarboxylic acid transporter 1 (MCT1, SLC16A1) and supports MM survival and proliferation. Additionally, BSG is implicated in response to treatment with immunomodulatory drugs (thalidomide and its derivatives). We investigated the role of single nucleotide polymorphisms (SNPs) in the gene coding for BSG and SLC16A1 in MM. Following an in silico analysis, eight SNPs (four in *BSG* and four in *SLC16A1*) predicted to have a functional effect were selected and analyzed in 135 MM patients and 135 healthy individuals. Alleles rs4919859 C, rs8637 G, and haplotype CG were associated with worse progression-free survival (*p* = 0.006, *p* = 0.017, *p* = 0.002, respectively), while rs7556664 A, rs7169 T and rs1049434 A (all in linkage disequilibrium (LD), *r*^2^ > 0.98) were associated with better overall survival (*p* = 0.021). Similar relationships were observed in thalidomide-treated patients. Moreover, rs4919859 C, rs8637 G, rs8259 A and the CG haplotype were more common in patients in stages II–III of the International Staging System (*p* < 0.05), while rs8259 A correlated with higher levels of β-2-microglobulin and creatinine (*p* < 0.05). Taken together, our results show that *BSG* and *SLC16A1* variants affect survival, and may play an important role in MM.

## 1. Introduction

Multiple myeloma (MM) is the second most common human haematologic malignancy. It is characterized by presence of atypical plasma cells (myeloma cells) in the bone marrow, impaired immunoglobulin production, and presence of monoclonal protein in serum and urine [1]. The number of new MM cases is estimated at ~20,000 per year in the United States, and this number is forecast to rise above 30,000 by 2032 [2].

Basigin (BSG), also known as CD147 and extracellular matrix metalloproteinase inducer (EMMPRIN), is a transmembrane glycoprotein and a member of the immunoglobulin superfamily [3]. It is widely expressed on many cells and also carries the Ok blood group in humans [4]. BSG is of major importance in myeloma cells, due to its participation in the transport of energy metabolism products, most importantly lactate anions. Lactates are produced in the glycolytic pathway and need to be removed from the cell because they decrease intracellular pH [5]. Lactate production is increased in cancer cells, as their reliance on the glycolytic pathway for energy production is much higher than that of normal cells—this is known as the Warburg effect. BSG is essential for lactate transport, although it is proteins of the monocarboxylate transporter (MCT) family that are directly responsible for it. MCTs are dependent on BSG as they are bound to it in the cell membrane, and BSG also functions as a chaperone for MCT1 and MCT4. This tight association is best seen by the fact that an MCT inhibitor was found to actually target BSG directly [3,6]. BSG overexpression was shown for many tumours, and has also been proved in multiple myeloma. Additionally, it has been demonstrated that an increase in BSG expression accompanies MM progression [7]. It was also found that MCT1 and MCT4 are overexpressed in MM, but that only MCT1 downregulation leads to decreased myeloma cell proliferation [5]. Other studies showed increased lactate export in myeloma cells, and a correlation of *BSG* gene expression with key regulators of Warburg effect, further confirming the role of BSG in lactate transport in MM [5,8].

A recent study showed that BSG is also implicated in MM treatment with immunomodulatory drugs (IMiDs), i.e., thalidomide and its derivatives [9]. IMiDs are known to exert their effect by binding to a protein called cereblon (CRBN) [10]. CRBN seems to promote maturation and activations of BSG and MCT1 through a chaperone-like mechanism. This process is independent of ubiquitination of proteins Ikaros and Aiolos, in which CRBN is involved, and which was shown earlier to lead to myeloma cell cycle arrest. This chaperone-like mechanism is abrogated following IMiD therapy, leading to accumulation of lactate in myeloma cells [9]. Additionally, another study showed that *BSG* mRNA expression is higher in patients responding to IMiD treatment than in non-responders [11].

These findings prompted us to analyse whether *BSG* and *SLC16A1* (*MCT1*) gene polymorphism might affect risk, survival, or outcome of treatment in multiple myeloma patients. In our previous studies, we showed that single nucleotide polymorphisms (SNPs) located in genes associated with IMiD metabolism do affect response to therapy [12,13]. In the present work, following an in silico analysis using the National Institute of Environmental Health Sciences SNP Function Prediction tool, we picked SNPs located within the *BSG* and *SLC16A1* genes that had minor allele frequency (MAF) in European populations higher than 0.15, and that were expected to have a functional effect [14]. Using the above criteria, eight SNPs were selected: *BSG* rs4919859—located in a potential transcription factor binding site, *BSG* rs8259—located in a potential microRNA binding site, *BSG* rs4682—located in an exonic splicing enhancer/silencer (ESE/ESS), *BSG* rs8637—located in an ESE/ESS and a potential microRNA binding site, *SLC16A1* rs9429505—located in a potential microRNA binding site, *SLC16A1* rs7556664—located in a potential transcription factor binding site, *SLC16A1* rs7169—located in a potential microRNA binding site and *SLC16A1* rs1049434—a missense Asp to Glu mutation.

## 2. Materials and Methods

### 2.1. Patients and Controls

The study included a group of 135 Polish MM patients and 135 healthy blood donors that served as controls. The group was also investigated in our previous study on *CTNNB1* (β-catenin) and *CRBN* variants; detailed information is included there [12]. In brief, the group of patients consisted of 70 males and 65 females, median age on diagnosis was 61 years. Among patients, 35% were in stage I, 34% in stage II, and 31% in stage III of the disease, according to the International Staging System (ISS) criteria. 74.1% were administered thalidomide as part of the first-line treatment, mostly together with cyclophosphamide and dexamethasone.

### 2.2. Genotyping

DNA was extracted from samples of peripheral blood taken on EDTA using Maxwell 16 blood DNA purification kit (Promega Corp., Madison, WI, USA) or silica membranes (Qiagen, Hilden, Germany), following the recommendations of the manufacturers. *BSG* and *SLC16A1* polymorphic variants were determined using the Taqman (Thermo Fisher, Waltham, MA, USA) and LightSNiP (TIB MOLBIOL, Berlin, Germany) assays. PCR was performed on a LightCycler 480 II device (Roche Diagnostics, Rotkreuz, Switzerland), according to the manufacturer’s recommendations.

### 2.3. Statistical Analysis

Linkage disequilibrium and Hardy-Weinberg equilibrium analyses were performed with the Haploview 4.2 software [15]. The null hypothesis that there is no difference between allele and genotype frequencies between patients and controls was tested with the Fisher’s exact test [16]. Survival was assessed using the Kaplan-Meier method, and associations with clinical parameters were calculated using the *U* Mann-Whitney test. Both of these analyses were performed with the real statistics resource pack for Microsoft excel 2013 (version 15.0.5023.1000, Microsoft, Redmont, Washington, DC, USA). *p* < 0.05 were considered statistically significant, and those between 0.05 and 0.10 as indicative of a trend. Genotypes were tested for deviations from Hardy-Weinberg equilibrium using the χ^2^ test.

## 3. Results

### 3.1. *BSG* and *SLC16A1* Allele and Genotype Frequencies

**Figure 1:**
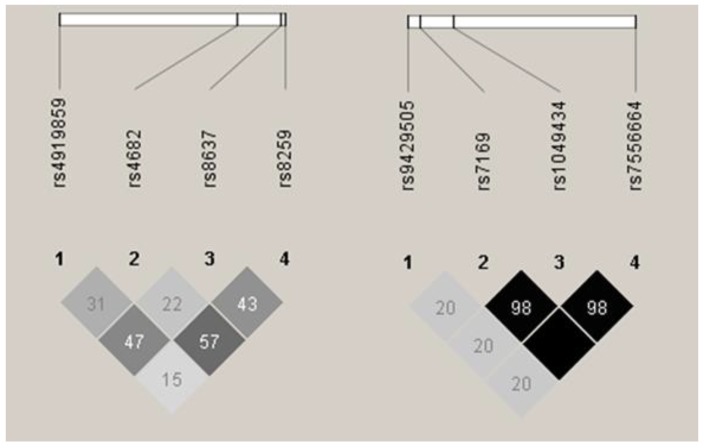
Linkage disequilibrium (LD) between single nucleotide polymorphisms (SNPs) under study. Basigin (*BSG)* SNPs are shown to the left, and monocarboxylic acid transporter 1 (*SLC16A1,* MCT1) to the right. Darker colour shows higher *r*^2^ values, while the value shown inside the squares is *r*^2^ × 10^2^. Results as presented by the Haploview 4.2 software [15].

Patients and healthy donors were genotyped for all eight SNPs, and allele and genotype frequencies were calculated for both MM patients and controls. Frequencies for all the SNPs were in accordance with Hardy-Weinberg equilibrium in both groups. Linkage disequilibrium (LD) analysis revealed that all the *BSG* SNPs were in low-to-medium LD (*r*^2^ range between 0.15 and 0.57, see Figure 1), while three of the four *SLC16A1* SNPs (rs7169, rs1049434, rs7556664) were in perfect or near perfect LD (*r*^2^ ≥ 0.98, see Figure 1). Because of this, rs7556664 was used as a tag SNP for rs7169 and rs1049434, and no further analyses were performed for the two latter SNPs.

We did not observe any statistically significant difference in allele distribution between MM patients and healthy controls in any of the SNPs tested. Distribution of genotypes in patients and controls is presented in Table 1.

### 3.2. Associations of *BSG* and *SLC16A1* Polymorphism with Survival

**Figure 2:**
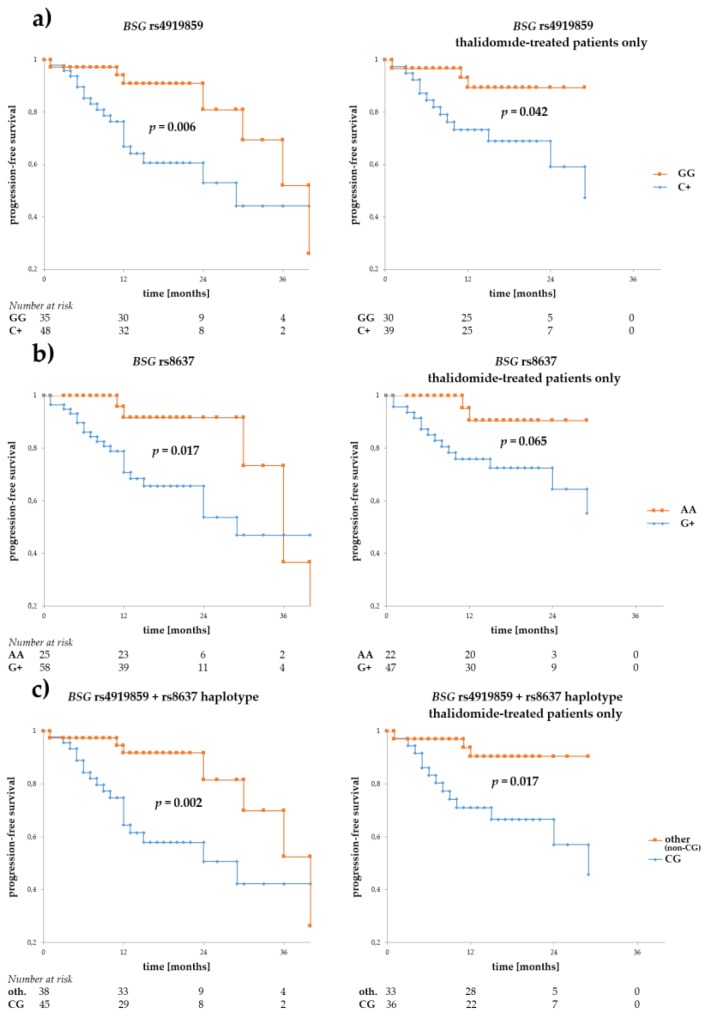
Progression-free survival in multiple myeloma (MM) patients with respect to *BSG* SNPs. Panels (**a**,**b**) show survival for rs4919859 C and rs8637 G, respectively. The two SNPs, located in a presumed transcription factor and microRNA binding sites, are in low/medium LD. Given their similar effect on progression-free (PFS), an rs4919859 C and rs8637 G (or CG, in short) haplotype was also tested for association with PFS, and is shown in panel (**c**). Curves to the left show PFS calculated for the entire group of patients, while those to the right show PFS calculated for the subgroup of thalidomide-treated patients. The numbers at risk show numbers of patients in the risk set (i.e., patients without disease progression, a number used to calculate survival) at a given time point on the *x*-axis. Survival was calculated using the Kaplan-Meier method and the real statistics resource pack for Microsoft excel 2013 (Microsoft).

We performed survival analyses for the SNPs regarding both progression-free (PFS) and overall survival (OS), in the whole MM patient group, and in a subgroup of patients treated with thalidomide (first-line treatment), constituting 74.1% of all patients. The analysis showed that alleles rs4919859 C and rs8637 G were particularly associated with adverse PFS (*p* = 0.006 and *p* = 0.017, for the C and G allele, respectively) (see Figure 2). Similar relationship with rs4919859 C and rs8637 G were observed in a subgroup of patients treated with thalidomide (*p* = 0.042 and *p* = 0.065, for the C and G allele, respectively). Given the similarity of rs4919859 C and rs8637 G regarding PFS and their relatively high LD, we investigated the CG haplotype and found it to be an even better predictor of adverse PFS (*p* = 0.002 and *p* = 0.017, for all patients and the thalidomide-treated subgroup respectively). Furthermore, a trend towards better PFS was observed for the *BSG* rs8259 T allele, but not in the thalidomide-treated subgroup (*p* = 0.102, *p* = 0.203, for all patients and the thalidomide-treated subgroup respectively, see Figure 2). 

No statistically significant association was observed for either of the *BSG* rs4682 alleles. Additionally, no statistically significant associations were observed for the *BSG* SNPs regarding OS, although there was a trend for slightly better OS in patients with rs4682 C (*p* = 0.097 for all patients, and *p* = 0.090 for the thalidomide-treated subgroup), and those with rs8637 G (*p* = 0.092 for all patients).

**Figure 3:**
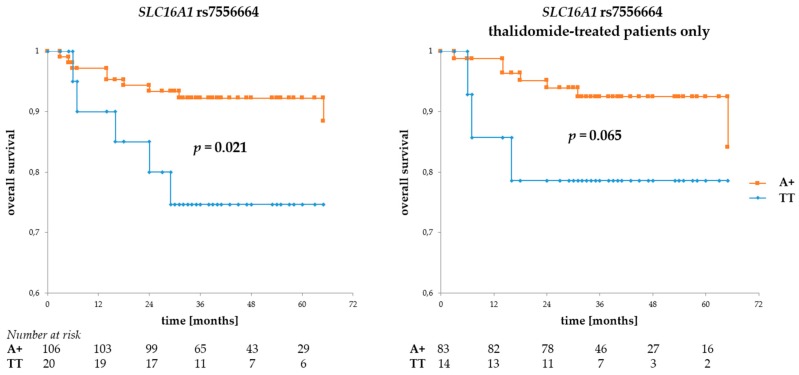
Overall survival in MM patients with respect to *SLC16A1* rs7556664 A. This SNP is in near-perfect LD with two other *SLC16A1* SNPs described in this study. Allele rs7556664 A (orange curve) also corresponds to rs1049434 A and rs7169 T; however, for the sake of simplicity, only rs7556664 A is shown in the figure above. The left panel shows OS calculated for the entire group of patients, while that to the right shows overall survival (OS) calculated for thalidomide-treated patients only. The numbers at risk show numbers of patients in the risk set (i.e., patients still living, a number used to calculate survival) at a given time point on the *x*-axis. Survival was calculated using the Kaplan-Meier method and the real statistics resource pack for Microsoft excel.

Regarding *SLC16A1* SNPs, the rs7556664 A allele (and, in consequence, rs7169 T and rs1049434 A) was associated with better OS (*p* = 0.021 for all patients and *p* = 0.065 for the thalidomide-treated subgroup, see Figure 3), but no such association was observed for either of the rs9429505 alleles. However, there was a trend for better PFS in patients with rs9429505 G (*p* = 0.078, for all patients). No statistically significant association regarding PFS was observed for rs7556664.

### 3.3. Influence of *BSG* and *SLC16A1* Polymorphism on Response to Treatment

Keeping in mind the importance of *BSG* and *SLC16A1* in CRBN-mediated response to IMiD therapy, we looked for associations between the SNPs under study and response to first-line treatment. Interestingly, we only observed a borderline significant association with *SLC16A1* rs9429505 (the G allele was more common in patients achieving complete or very good partial remission than in patients achieving only partial remission or no remission, *p* = 0.048), and a trend towards better response with *BSG* rs8259 (allele A was more common in patients achieving complete or very good partial remission, *p* = 0.082). Both were seen in patients administered the cyclophosphamide, thalidomide, dexamethasone (CTD) regimen.

### 3.4. BSG and SLC16A1 Polymorphism and Other Clinical Parameters

We compared the occurrence of *BSG* and *SLC16A1* variants in patients with various MM stages on diagnosis according to the international staging system (ISS). Interestingly, we found alleles rs4919859 C, rs8259 A, and rs8637 G and the CG haplotype (as defined in the Section 3.2*.* dealing with survival) to be more common in patients in stages II–III than in those in stage I (*p* = 0.024, *p* = 0.001, *p* < 0.001, *p* = 0.001, respectively). A similar, although not statistically significant, relationship was also observed for allele rs4682 C (*p* = 0.053). However, no association between ISS stages and *SLC16A1* variants was observed.

Regarding other clinical parameters, we found rs8259 A to be associated with higher β-2-microglobulin and creatinine levels (*p* = 0.022 and *p* = 0.017, respectively).

## 4. Discussion

In recent years, there have been many significant discoveries regarding the processes occurring during response to MM therapy with immunomodulatory drugs. First, CRBN was found to play a central role, and further studies showed importance of proteins Ikaros and Aiolos, as well as BSG and MCT1 [9,10,17]. Additionally, overexpressed BSG and MCT1 were demonstrated to be involved in energy metabolism in MM by the means of supporting the export of toxic lactate anions in myeloma cells [5,7].

Our present study aimed at elucidating the role played by genetic variability in *BSG* and *SLC16A1* (MCT1) in myeloma cells. Three SNPs located in the *SLC16A1* gene turned out to be in near-perfect LD; therefore, only one of them was chosen for further analysis (rs7556664). It should, however, be noted that the observed effect of rs7556664 could also be equally attributed to either rs7169 or rs1049434.

Of the eight SNPs under study, rs4682, rs4919859, rs7169, and rs7556664 have, to the best of our knowledge, never been studied before. *BSG* rs8637 was mentioned in the context of carotid atherosclerotic plaques, but the study found the SNP not to be associated with risk for this disease [18]. rs8259 was described in three different non-haematologic diseases in which allele T was associated with lower mRNA *BSG* expression and lower plasma levels of soluble BSG [19,20,21]. This appears to agree with our findings, as rs8259 T seems to positively affect progression-free survival.

*SLC16A1* rs1049434 (in near-perfect LD with rs7169 and rs7556664) was studied the most due to its exonic location, and the fact that it affects MCT1 protein structure [22,23,24,25]. It has been shown that the mutated variant (containing Glu in the protein sequence and T in the DNA sequence) exhibits increased lactate transport via SLA16A1, as compared to the wild type Asp/A [26]. This seems to be in line with our results showing that rs1049434 A is correlated with better survival (increased lactate transport is favourable for myeloma cells, and therefore detrimental for patient’s survival).

We do not know how the other SNPs described in this study affect BSG, but given their presumed functions (as described in the introduction), we expect them to affect *BSG* and *SLC16A1* expression (both on mRNA and protein level, depending on whether or not they are located in a potential transcription factor or miRNA binding site), or splicing (especially in the case of rs4682).

In the present study, we observed an interesting association with *BSG* and *SLC16A1* SNPs and progression-free and overall survival. These associations show that the selected *BSG* and *SLC16A1* alleles may influence survival in MM patients in general. However, it should be noted that some of the relationships (e.g., SLC16A1 SNPs and survival) cannot be ascertained definitively due to the relatively small size of one of the subgroups analysed. Furthermore, because many of the associations were no longer statistically significant after limiting the group to patients treated with thalidomide, we cannot decisively conclude that the various alleles affected CRBN-mediated response to thalidomide treatment. Indeed, it is possible that the observable effect might have been exclusively due to the *BSG* and *SLC16A1* alleles variously affecting BSG-governed lactate export. As described in the results section, we found correlations with response to thalidomide treatment, although only in the case of rs9429505 was the relationship statistically significant. This relative lack of correlations between *BSG* SNPs and response to treatment seems to be at odds with results of Bolomsky et al., as they showed that *BSG* mRNA expression was significantly higher in patients responding to IMiD therapy [11]. Interestingly though, a more recent study showed no association between *BSG* mRNA expression and response to IMiD treatment [27]. However, it should be noted that comparison of such studies might be elusive, as the exact therapies used (e.g., IMiD chosen, other drugs used together with IMiDs, dosages etc.) vary between studies. In addition, some studies suggest that CRBN-mediated response to IMiD is controlled largely in a post-translational manner [9,28]. Regardless of molecular explanations for the survival associations, it is interesting to note that Krönke et al. also reported an association between *BSG* expression levels and PFS, albeit only in patients with standard (and not high) cytogenetic risk [27].

In conclusion, our study shows that selected *BSG* and *SLC16A1* genetic variants may affect progression-free and overall survival in multiple myeloma, and correlate with various clinical parameters of MM, such as ISS stage, β-2-microglobulin and creatinine levels. These results should be further confirmed on larger cohorts of MM patients. More studies are necessary to elucidate the precise mechanism of these variants and the means by which they affect BSG/SLC16A1 levels and functions.

## Figures and Tables

**Table 1:** Distribution of *BSG* and *SLC16A1* genotypes in multiple myeloma (MM) patients (entire patient cohort and thalidomide-treated patients only) and healthy controls.

	MM Patients (All)	MM Patients (Thalidomide-Treated)	Controls		MM Patients	MM Patients (Thalidomide-Treated)	Controls
*BSG* rs4919859				*SLC16A1* rs9429505			
CC	15 (11.2%)	13 (13.1%)	19 (14.1%)	AA	77 (57.0%)	59 (59.0%)	67 (49.6%)
CG	65 (48.5%)	47 (47.5%)	54 (40.0%)	AG	51 (37.8%)	34 (34.0%)	60 (44.4%)
GG	54 (40.3%)	39 (39.4%)	62 (45.9%)	GG	7 (5.2%)	7 (7.0%)	8 (5.9%)
*BSG* rs4682				*SLC16A1* rs7169			
CC	3 (2.2%)	3 (3.0%)	4 (3.0%)	CC	22 (16.3%)	15 (15.0%)	23 (17.0%)
CT	45 (33.6%)	35 (35.4%)	34 (25.4%)	CT	63 (46.7%)	46 (46.0%)	58 (43.0%)
TT	86 (64.2%)	61 (61.6%)	96 (71.6%)	TT	50 (37.0%)	39 (39.0%)	54 (40.0%)
*BSG* rs8637				*SLC16A1* rs1049434			
AA	44 (32.8%)	32 (32.3%)	47 (34.8%)	AA	51 (37.8%)	39 (39.0%)	54 (40.0%)
AG	63 (47.0%)	45 (45.5%)	61 (45.2%)	AT	62 (45.9%)	46 (46.0%)	58 (43.0%)
GG	27 (20.2%)	22 (22.2%)	27 (20.0%)	TT	22 (16.3%)	15 (15.0%)	23 (17.0%)
*BSG* rs8259				*SLC16A1* rs7556664			
TT	77 (57.0%)	57 (57.0%)	78 (57.8%)	AA	50 (37.0%)	39 (39.0%)	54 (40.0%)
TA	49 (36.3%)	35 (35.0%)	49 (36.3%)	AT	63 (46.7%)	46 (46.0%)	58 (43.0%)
AA	9 (6.7%)	8 (8.0%)	8 (5.9%)	TT	22 (16.3%)	15 (15.0%)	23 (17.0%)

Numbers of patients and controls analysed for various SNPs are slightly different. One person was not included in calculations for rs4919859, rs4682, rs8637 (patients) and rs4682 (controls).
